# Continuous personalized cerebrovascular reactivity-based physiologic metrics in neurocritical care: a narrative review of the current landscape, limitations, and future directions

**DOI:** 10.3389/fphys.2025.1582813

**Published:** 2025-05-06

**Authors:** Kevin Y. Stein, Logan Froese, Rakibul Hasan, Amanjyot Singh Sainbhi, Nuray Vakitbilir, Tobias Bergmann, Abrar Islam, Noah Silvaggio, Mansoor Hayat, Frederick A. Zeiler

**Affiliations:** ^1^ Biomedical Engineering, Price Faculty of Engineering, University of Manitoba, Winnipeg, MB, Canada; ^2^ Max Rady College of Medicine, Rady Faculty of Health Sciences, University of Manitoba, Winnipeg, MB, Canada; ^3^ Department of Clinical Neuroscience, Karolinska Institutet, Stockholm, Sweden; ^4^ Department of Human Anatomy and Cell Science, Rady Faculty of Health Sciences, University of Manitoba, Winnipeg, MB, Canada; ^5^ Section of Neurosurgery, Department of Surgery, Rady Faculty of Health Sciences, University of Manitoba, Winnipeg, MB, Canada; ^6^ Pan Am Clinic Foundation, Winnipeg, MB, Canada

**Keywords:** personalized medicine, cerebrovascular reactivity, cerebral perfusion pressure optimum, mean arterial pressure optimum, bispectral index optimum, individualized intracranial pressure thresholds

## Abstract

Over the past several decades, significant progress has been made in our ability to achieve guideline-based cerebral physiologic targets for the management of moderate-to-severe traumatic brain injury (TBI). However, despite these advancements, there has been limited improvement in the long-term outcomes associated with this condition. It has been suggested that this is in part due to the generalized approach of current Brain Trauma Foundation guidelines. It has been demonstrated that significant heterogeneity in cerebral physiologic response to TBI exists between patients, and that it involves highly dynamic physiologic mechanisms which vary across a patient’s time in the ICU. Therefore, an individualized management approach, that accounts for individual phenotype, injury heterogeneity, and the dynamic nature of cerebral physiology, is urgently needed. Recently, multiple personalized physiologic metrics, based on cerebrovascular reactivity optimization, have been proposed as potential tools to help address this increasingly important issue. These include the cerebral perfusion pressure optimum (CPPopt), mean arterial pressure optimum (MAPopt), bispectral index optimum (BISopt), and individualized intracranial pressure (iICP) thresholds. These metrics aim to shift neurocritical care management from static, population-based targets to dynamic, personalized targets that are tailored to a patient’s real-time cerebral physiologic needs. In this narrative review, we will cover the topic of continuously derived cerebrovascular reactivity-based personalized physiologic metrics in neurocritical care, including the current states of the various existing techniques, their limitations, and future directions.

## Introduction

Neurocritical conditions, such as traumatic brain injury (TBI), are a leading cause of death and disability globally (Zhong et al., 2025). The devastating effects of such neurocritical conditions are not only a result of the immediate structural damage that occurs at the time of the initial insult, termed primary brain injury, but also the downstream derangements in cerebral physiology that occur in response to this initial damage. Such secondary brain injury results in ongoing neuronal death in the days, months, or even years following the cerebral injury and significantly impedes recovery (Maas et al., 2017). Since very little can be done to reverse primary brain injury, neurocritical management is almost exclusively limited to minimizing secondary injury.

Due to the fixed volume of the cranial cavity, increases in intracranial contents, such as from brain bleeding or swelling, can result in significant elevations in intracranial pressure (ICP). This can pose significant risks to the cerebral environment, such as tissue herniation and disruptions in cerebral blood flow (CBF). The latter can be explained by the inherent relationship that exists between ICP and the pressure driving CBF, termed cerebral perfusion pressure (CPP): CPP = arterial blood pressure (ABP) – ICP. Normally, CBF is kept relatively constant by various cerebral autoregulatory mechanisms; however, these mechanisms often become impaired following TBI, exposing the brain to pressure-passive changes in CBF (Toth et al., 2016).

Given the brain’s significant metabolic demand–accounting for approximately 25% of basal metabolism despite comprising only about 2.5% of the body’s weight–and its limited capacity to store energy, cerebral tissue is highly vulnerable to ischemic damage (Lee et al., 2000). Therefore, careful monitoring and timely therapeutic interventions are essential to prevent critically elevated ICP or insufficient CPP when managing neurocritical patients. Since cerebral hyperperfusion can also be harmful to the brain, through blood-brain barrier breakdown (Fantini et al., 2016), grossly elevated CPP should also be avoided.

Extensive literature has demonstrated that intracranial hypertension contributes significantly to poor outcomes in head injured patients and that aggressive ICP/CPP management is associated with better recovery (Alali et al., 2013; Chesnut et al., 2012; Farahvar et al., 2012; Gerber et al., 2013; Talving et al., 2013). Therefore, standard care for moderate-to-severe TBI, outlined by the Brain Trauma Foundation (BTF) guidelines (Carney et al., 2017; Hawryluk et al., 2019), primarily revolves around an ICP/CPP-directed approach. The monitoring of ABP and ICP lies at the cornerstone of such management, with ABP typically monitored using an arterial blood line and pressure transducer and ICP monitored invasively using either an external ventricular drain (EVD) or an implantable microtransducer device (Raboel et al., 2012). In recent years, non-invasive ICP monitoring modalities have also been developed; however, invasive monitoring remains the most accurate and thus the recommended modality in the neurocritical care setting (de Moraes et al., 2023).

Current BTF guidelines recommend therapeutically maintaining ICP below a threshold of 20 or 22 mmHg and CPP within a target range of 60–70 mmHg (Carney et al., 2017; Hawryluk et al., 2019). However, it should be noted that there currently exists a level of ambiguity around whether this 60–70 mmHg CPP range is truly a target or rather a minimum threshold. A variety of therapeutic options are available to help clinicians achieve these targets, including induced hyperventilation, infusion of hyperosmolar agents, administration of sedatives, analgesics, and paralytics, drainage of cerebrospinal fluid, decompressive craniectomy, and administration of corticosteroids (Rangel-Castilla et al., 2008a). However, it should be noted that due to the inherent risks associated with ICP lowering therapeutics, clinicians must balance the importance of achieving guideline recommended physiologic targets with the iatrogenic risks of such therapies (Carney et al., 2017; Freeman, 2015).

### Shortcomings of current practices

Over the past several decades, our capabilities to achieve guideline-based physiologic targets have drastically improved; however, despite such advances, the poor outcomes associated with moderate-to-severe TBI have remained relatively unchanged (Maas et al., 2017; Steyerberg et al., 2019; Donnelly et al., 2019). This discrepancy has been attributed to the to the fact that these guideline-based targets were identified through population-based analyses that evaluated the association between grand-averaged cerebral physiology and long-term outcomes (Anonymous, 2000), and therefore, fail to account for individual-phenotype, injury heterogeneity, and the dynamic nature of cerebral physiology (Stocchetti et al., 2017). This is further supported by recent studies that have suggested that existing population-based prognostic models account for less than half of the outcome variance seen in TBI (Zeiler et al., 2021a; Dijkland et al., 2021; Steyerberg et al., 2008).

The cerebral physiologic response to brain trauma varies drastically between individuals (Stocchetti et al., 2017; Zeiler et al., 2021a; Le Roux et al., 2014; Zeiler et al., 2020a; Okonkwo et al., 2017; Depreitere et al., 2021). A variety of patient-specific factors influence how one’s brain responds to neurological insult. For example, older patients have been shown to have worse long-term outcomes than younger patients (Czosnyka et al., 2005). Sex-related differences also exist. A 2008 study by Czosnyka et al. found that females tended to have higher mortality rates than males, but only when looking at patients who were less than 50 years of age (Czosnyka et al., 2008). More recently, Åkerlund et al. were even able to distinguish six distinct TBI endotypes using various demographic, physiologic, and clinical factors (Åkerlund et al., 2022). Moreover, the authors demonstrated that complementation of existing prognostic models with these endotypes drastically improved outcome prediction capabilities. Genetic factors have also been shown to contribute to post-TBI injury-response variability. Genetic polymorphisms in several genes, affiliated with a wide range of functions such as neural repair, vascular response, inflammation, neurotransmission, and blood-brain barrier maintenance, have been implicated with long-term outcomes post-TBI (Gomez et al., 2021a; Zeiler et al., 2021b; Lakshmipathy et al., 2024).

Furthermore, the effectiveness of current therapeutic options have been shown to differ significantly between various patient subgroups (Zeiler et al., 2021a; Froese et al., 2020a). Recent literature has also shown that a significant amount of cerebral physiologic insult burden does not respond to these therapeutics (Donnelly et al., 2019; Froese et al., 2020b; Zeiler et al., 2019a; Froese et al., 2021a; Tang et al., 2015; Dias et al., 2014; Timofeev et al., 2008; Wettervik et al., 2020). Therefore, it is vital for moderate-to-severe TBI management to move away from its current “one treatment fits all” paradigm and incorporate personalized approaches that can be tailored to the individual patient. A promising way forward is through the development of personalized therapeutic targets that are patient-specific and allow clinicians to address a patient’s individual physiologic needs. Moreover, since it is likely that optimal therapeutic interventions and targets not only vary from patient to patient, but also throughout a patient’s time in the intensive care unit (ICU), personalized targets that can be continuously derived at patient-bedside are particularly promising.

### Cerebral autoregulation

CPP represents the driving pressure behind CBF. Since both ABP and ICP are non-static parameters, CPP is subject to frequent fluctuations. Without any protective mechanisms, these fluctuations would expose the brain to pressure-passive changes in CBF, resulting in hypoperfusion during low systemic pressures and hyperperfusion during high systemic pressures (Armstead, 2016). However, through a critical physiological mechanism termed cerebral autoregulation, cerebral vessels are able to self-regulate their tone in response to changes in CPP, as well as neurogenic, myogenic, and metabolic factors, in order to maintain a relatively stable CBF despite changes in systemic pressure (Rangel-Castilla et al., 2008b). This process is crucial for protecting the brain from both hypo- and hyper-perfusion related injury, and thus has become one of the most explored continuous cerebral physiologies in neurocritical care.

**FIGURE 1:**
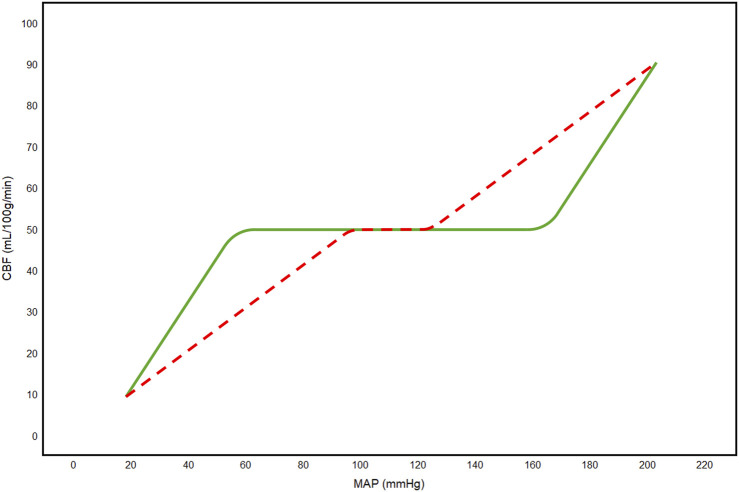
Lassen autoregulatory curve. Green line represents the curve in the physiologic state. Red dashed line represents the curve in a pathological state. CBF, cerebral blood flow; MAP, mean arterial pressure; mmHg, millimeters of mercury.

Traditionally, cerebral autoregulation, and its limits, has been understood through the lens of the Lassen autoregulatory curve (Armstead, 2016; Lassen, 1959), shown in Figure 1, which suggests that, in the non-pathological state, cerebral autoregulation is able to maintain a constant CBF between mean arterial pressures (MAP) of approximately 60 and 160 mmHg, beyond which CBF becomes deranged. However, it should be noted that Lassen’s model was developed using data averaged from multiple individuals under various pharmacologic and pathologic conditions. As a result, some have argued that the Lassen curve overstates the brain’s ability to maintain stable CBF and that cerebral autoregulation is more pressure-passive and variable than originally thought (Brassard et al., 2021). Furthermore, there is experimental evidence suggesting that the autoregulatory plateau is much narrower (∼5–10 mmHg) than Lassen originally proposed (Tan, 2012), that autoregulatory responses are asymmetric, being more effective during increases than decreases in pressure (Numan et al., 2014), and that the limits of autoregulation vary widely between individuals (Drummond, 1997). Regardless, this autoregulatory mechanism often becomes impaired in neurological conditions such as TBI, significantly reducing the range of systemic blood pressures over which the mechanism is capable of keeping CBF constant (Toth et al., 2016; Lassen, 1959). This can result in pressure-passive state where CBF fluctuates in accordance with systemic pressure, exposing the brain to further secondary brain injury.

The intactness of cerebral autoregulation can be estimated using flow-based indices, which leverage transcranial Doppler (TCD) ultrasonography measured cerebral blood flow velocity (CBFV). This includes the Mean Flow Index (Mx) and the Systolic Flow Index (Sx), which are calculated as the correlation between CPP and mean or systolic CBFV, respectively (Czosnyka et al., 1996). However, the reliance of these flow-based indices on continuous TCD monitoring, which is technically challenging due to probe fixation, operator dependence, and susceptibility to movement artifacts, makes them unpractical for long-term monitoring in the clinical setting.

### Cerebrovascular pressure reactivity

A closely related, but distinct, mechanism is cerebrovascular pressure reactivity, which we will, for simplicity, just refer to as cerebrovascular reactivity (CVR) throughout the rest of this review. CVR refers to the ability of the cerebral vasculature to self-regulate smooth muscle tone in response to changes in transmural pressure and represents an important component of cerebral autoregulation (Budohoski et al., 2012). Continuous monitoring of CVR at the patient bedside is possible through the calculation of a Pearson correlation between a surrogate for pulsatile cerebral blood volume (i.e., ICP) and a driving pressure of CBF (i.e., MAP or CPP) (Budohoski et al., 2012; Zeiler et al., 2017a; Zeiler et al., 2020b; Zweifel et al., 2008). Generally, a negative or near zero correlation coefficient indicates intact reactivity (Sorrentino et al., 2012). This is because when CVR is intact, it counteracts changes in driving pressure, resulting in either no change in CBF or small opposing changes. When CVR is impaired, typically a positive correlation coefficient is observed, since CBF will directly mirror changes in driving pressure.

**FIGURE 2:**
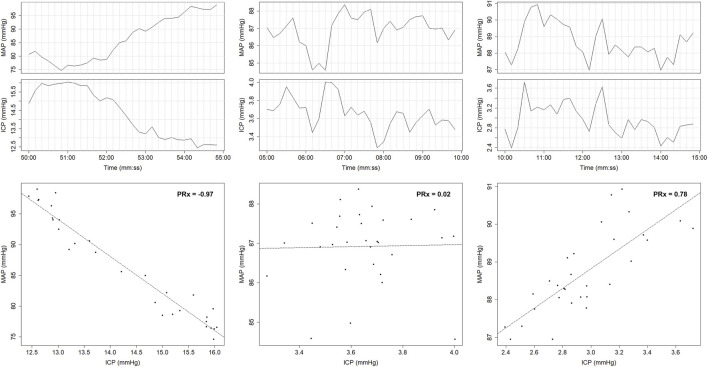
PRx calculated as the Pearson correlation coefficient between 30 consecutive 10-s averaged windows of ICP and MAP. Time trends display the corresponding 300-s windows of 10-s averaged ICP and MAP data used to generate the correlation coefficients. ICP, intracranial pressure; MAP, mean arterial pressure; mmHg, millimeters of mercury; PRx, pressure reactivity index. University of Manitoba Health Research Ethics Board approval for generation of this image - H2024:266.

Several indices can be used to evaluate CVR; however, ICP-based indices are particularly convenient due to the widespread use of ICP monitoring in the neurocritical care setting (Zeiler et al., 2020c). The pressure reactivity index (PRx), which measures the Pearson correlation between slow vasogenic waves of ICP and MAP (Czosnyka et al., 1997), has been the most widely studied. Examples of PRx derivation are presented in Figure 2. However, other ICP-based indices also exist, such as the pulse amplitude index (PAx), which evaluates the correlation between the fundamental pulse amplitude of ICP (AMP) and MAP (Radolovich et al., 2011), and the RAC index, which assesses the correlation (R) between AMP (A) and CPP (C) (Zeiler et al., 2018a).

Although ICP-based indices offer a practical and reliable method for assessing CVR at the patient bedside, their dependency on invasive ICP monitoring restricts their use in the broader ICU population, where ICP monitoring is often not clinically indicated. To address this limitation, the cerebral oximetry index (COx_a) has been proposed as an entirely non-invasive alternative to ICP-based CVR indices (Brady et al., 2010a). COx_a is calculated using near-infrared spectroscopy (NIRS)-based regional cerebral oxygen saturation (rSO_2_) as a surrogate for cerebral blood volume, and MAP, which can be measured non-invasively using finger cuff technology, as a surrogate for driving pressure (Gomez et al., 2021b; Gomez et al., 2020). Studies have demonstrated that COx_a provides comparable representations of CVR to PRx (Zeiler et al., 2017a; Zeiler et al., 2017b; Zweifel et al., 2010; Mathieu et al., 2020; Gomez et al., 2023), and even captures aspects of the Lassen autoregulatory curve (Brady et al., 2010a; Sainbhi et al., 2021; Lee et al., 2012). This positions COx_a as a potential tool for non-invasively monitoring CVR in non-TBI populations. However, COx_a remains a problematic index due to the limited penetration depth of NIRS, restricting assessment to superficial cortical regions, and the tendency of NIRS devices to output low-frequency, non-pulsatile signals (usually ∼1–2 Hz) (Sainbhi et al., 2023). This often results in limited variability in rSO_2_ and COx_a values near zero, thereby complicating its interpretation. It should be noted that a variant form that uses CPP in place of MAP also exists (COx); however, due to its use of CPP, it cannot be measured non-invasively.

Over the past decade, impaired CVR has been shown to be associated with secondary brain injury following TBI, with patients exhibiting such impairments for significant portions of their ICU stays (Donnelly et al., 2019; Sorrentino et al., 2012; Zeiler et al., 2020c; Czosnyka et al., 1997; Zeiler et al., 2018b; Zeiler et al., 2019b). Numerous studies have also established a strong association between impaired CVR and poor long-term outcomes post-TBI (Donnelly et al., 2019; Zeiler et al., 2020c; Zeiler et al., 2019b), with both mean values of CVR surrogate metrics and duration of impairment during the acute phase having been demonstrated to be significantly associated with 6-month outcomes (Sorrentino et al., 2012; Zeiler et al., 2019b; Adams et al., 2017; Bennis et al., 2020). These associations have also been demonstrated in the pediatric population as well (Appavu et al., 2021; Agrawal et al., 2025; Smith et al., 2023). The literature highlights the importance of preserving intact CVR, and has even led current neurocritical care consensus statements to include CVR monitoring as part of their recommendations for moderate-to-severe TBI management (Le Roux et al., 2014; Czosnyka et al., 2014). However, it should be noted that these recommendations remain weak due to insufficient evidence demonstrating the clinical significance of preserving intact CVR. Furthermore, no specific indices or thresholds have been definitively identified as optimal for guiding clinical management.

CVR optimization, which should theoretically help minimize secondary brain insult, has been used as the basis for the development of various personalized cerebral physiologic targets. Currently, a total of four personalized targets, that can be derived continuously (or have the potential to be), have been developed. These include the cerebral perfusion pressure optimum (CPPopt), mean arterial pressure optimum (MAPopt), bispectral index optimum (BISopt), and individualized intracranial pressure (iICP) thresholds. All four personalized targets use the relationship between some cerebral physiologic metric and CVR to determine patient-specific treatment targets that theoretically optimize CVR status. It should be noted that, while an extensive amount of literature on CPPopt exists, the remaining three personalized targets are currently only supported by preliminary data from a limited number of research groups. Throughout the rest of this paper, we will provide a general overview of all four existing personalized cerebral physiologic targets.

## Cerebral perfusion pressure optimal (CPPopt)

In 2001, Czosnyka et al. conducted a groundbreaking study investigating the relationship between CVR and cerebral physiology (Czosnyka et al., 2001). They found that CPP and Mx exhibited a U-shaped relationship, with both low and high CPP being associated with impaired CVR. One year later, based on this observation, Steiner et al. hypothesized that it would be feasible to identify a patient-specific CPP value where CVR is most optimal, which they termed CPPopt (Steiner et al., 2002). To achieve this, a patient’s CPP data was divided into 5 mmHg bins and an average PRx was calculated for each bin. The bins of data were then plotted using error bars. When a U-shaped curve was observed, the CPP bin with the lowest average PRx was manually identified as the patient’s CPPopt.

In a cohort of 114 head-injured patients, the authors were able to identify a CPPopt in 60% of patients (Steiner et al., 2002). However, in 27% of patients without an identifiable CPPopt, a partial ascending or descending curve was observed, suggesting that a CPPopt could potentially have existed outside of the patient’s available data range. Among patients with an identifiable CPPopt, a strong correlation between the absolute deviation of mean CPP from CPPopt (ΔCPPopt) and outcome (r = −0.51, p < 0.00001) was observed, suggesting that having a mean CPP value further from one’s identified CPPopt is associated with worse outcomes. Both CPP above (r = 0.53, p < 0.001) and below (r = −0.40, p < 0.05) CPPopt were linked to poorer outcomes, indicating that both inadequate and excessive CPP are detrimental to one’s cerebral autoregulatory status.

The feasibility of deriving CPPopt using this methodology was later validated in both subarachnoid hemorrhage (SAH) and intracerebral hemorrhage (ICH) patients (Bijlenga et al., 2010; Rasulo et al., 2012; Santos et al., 2011). In a 2010 study by Bijlenga et al., it was observed that, when comparing CPPopt curves during baseline and during episodes of vasospasm, CPPopt values were generally higher during vasospasm. This suggested that CPPopt is not a static parameter, but rather changes overtime depending on a patient’s cerebrovascular state (Bijlenga et al., 2010).

### Continuous CPPopt derivation

**FIGURE 3:**
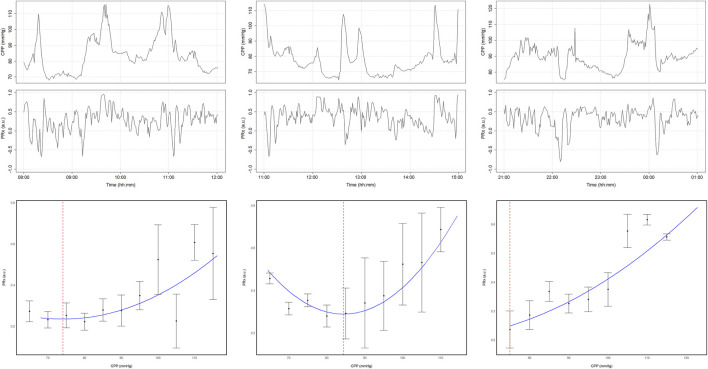
Example CPPopt curves and their respective 4-h windows of CPP and PRx data. All three examples were generated from the same patient’s dataset. Error bars represent 95% confidence intervals for 5 mmHg CPP bins. The blue curves represent the fit parabolic curves. The vertical red dashed lines represent the identified CPPopt values. The time trends present the minute-by-minute CPP and PRx data for the 4-h windows used to generate each curve. a.u., arbitrary units; CPP, cerebral perfusion pressure; CPPopt, cerebral perfusion pressure optimum; mmHg, millimeters of mercury; PRx, pressure reactivity index. University of Manitoba Health Research Ethics Board approval for generation of this image - H2024:266.

The original CPPopt methodology laid out by Steiner et al. suffered from two major limitations that prevented any potential clinical application: its dependency on completed data recordings, preventing real-time derivation, and its reliance on manual inspection of the CPP vs. PRx relationship. In 2012, Aries et al. addressed these shortcomings by developing an automated, continuously updating CPPopt algorithm and demonstrating the feasibility of continuously deriving CPPopt (Aries et al., 2012a). Using the same general principles laid out by Steiner et al., the authors leveraged an automated curve fitting method and a sliding 4-h time window to calculate CPPopt on a minute-by-minute basis. For a more detailed summary of this algorithm, the interested reader is referred to the original article. Example curves generated from various 4-h windows of data are presented in Figure 3.

When applied to a cohort of 327 severe TBI patients, CPPopt was identifiable, on average, during 55% of a patient’s recording period. It was found that the deviation between continuously updating median CPP and CPPopt was significantly associated with patient outcomes (Χ^2^ = 45, p < 0.001) and demonstrated a more robust association with outcomes than either guideline-based CPP.

Dias et al. later demonstrated the feasibility of real-time CPPopt-targeted management (Dias et al., 2015). In their study, CPPopt was continuously derived at the bedsides of 18 severe TBI patients, using the algorithm developed by Aries et al., and used to guide CPP management whenever possible. A CPPopt was available in approximately 59% of the total recording time. When patients were dichotomized based on 6-month Glasgow Outcome Scale (GOS) score into those with adverse (GOS < 3) and favorable (GOS ≥ 3) outcomes, the mean ΔCPPopt was found to be of greater magnitude in the adverse group (p = 0.04).

Despite the significant advancements introduced by the Aries algorithm, key limitations remained. The algorithm suffered from a relatively low derivation yield, averaging around 55%, and significant CPPopt output instability, with values often fluctuating drastically over short periods. In 2014, Depreitere et al. attempted to address these limitations by leveraging a multi-window weighted approach (Depreitere et al., 2014). Instead of solely using a fixed 4-h window, the authors applied the general methodology to time windows of 1, 2, 4, 6, 8, 12, and 24 h. Plots were then weighed based on two key criteria: the better a U-shaped curve could be fit (as defined by R^2^ heuristic) and the lower the CVR index at CPPopt, the greater the weight. A weighted average of the plots was then used to determine the final CPPopt value. This process was done on a minute-by-minute basis. This algorithm is included in the widely used Intensive Care Monitoring “Plus” (ICM+) software (Cambridge Enterprise Ltd., Cambridge, UK, http://icmplus.neurosurg.cam.ac.uk) as the Optimal Flex method.

When applied to a cohort of 180 patients, this multi-window weighted methodology was able to identify a CPPopt in 95% (interquartile range [IQR]: 90%–97%) of monitoring time, a drastic improvement over the previous work. Regarding outcome, it was found that the proportion of time spent within 5 mmHg of CPPopt (within the range from 5 mmHg below CPPopt to 5 mmHg above CPPopt) was statistically higher for survivors than non-survivors (25.6% vs. 19.6%, p = 0.01). This finding was mirrored by a later study by Young et al. which found that time spent within 10 mmHg of CPPopt was greater in survivors than non-survivors (p = 0.02) (Young et al., 2016).

Three years later, Liu et al. further refined this multi-window weighted methodology by implementing a more sophisticated windowing strategy that incorporated 36 overlapping windows, ranging between 2 and 8 h in increasing 10-min increments, and an improved weighting system that favored shorter windows, lower curve fit error, and the presence of parabolic shape (Liu et al., 2017a). Additionally, the authors provided an in depth comparison between continuous CPPopt derivation using a multi-window weighted approach and the earlier fixed 4-h sliding window method. It was demonstrated that not only does the multi-window weighted approach substantially improve CPPopt derivation yield (p < 0.05) but also significantly enhance CPPopt stability, as observed as a reduction in the standard deviation (SD) of sample-to-sample differences (p < 0.05).

Later, Beqiri et al. introduced further refinements to this continuous CPPopt algorithm to improve its reliability and stability (Beqiri et al., 2023). Recognizing the challenges of the previous approaches, the authors implemented stricter curve-fitting criteria, increased the robustness of the weighting heuristics, and incorporated additional filtering steps to reduce false-positive CPPopt values caused by non-physiological variations in ICP and MAP. These refinements led to a significantly more stable CPPopt output (p < 0.0001), with fewer abrupt changes over short periods, while maintaining its ability to predict mortality. However, this came at the cost of a slightly reduced derivation yield.

**FIGURE 4:**
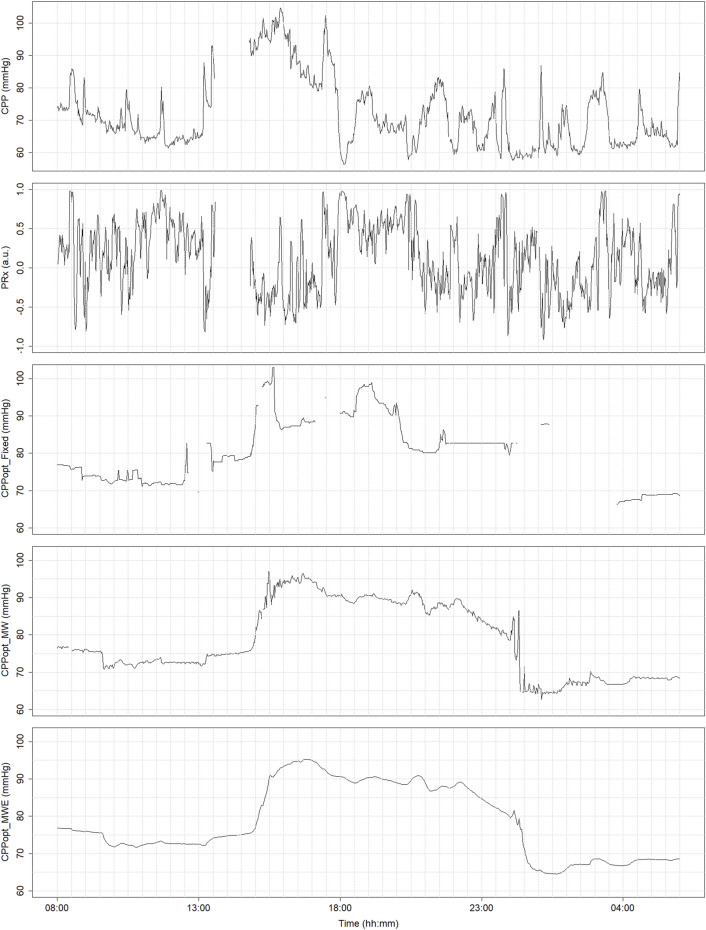
Time trends of continuously derived CPPopt using the 4-h fixed window method (Fixed), the multi-window weighted method (MW), and the enhanced multi-window weighted method (MWE). The time trends display data at a minute-by-minute resolution. a.u., arbitrary units; CPP, cerebral perfusion pressure; CPPopt, cerebral perfusion pressure optimum; mmHg, millimeters of mercury; PRx, pressure reactivity index. University of Manitoba Health Research Ethics Board approval for generation of this image - H2024:266.

CPPopt time trends generated using the various derivation methodologies mentioned here are presented in Figure 4. It is notable that, despite all of the methodologies aiming to measure the same physiologic mechanism, a considerable amount of variability exists between their outputs due to differences in parameter settings. For example, it is evident that the multi-window weighted methodologies result in superior CPPopt signal continuity compared to the fixed-window approach. The improved CPPopt stability of the Beqiri et al. methodology can also be observed. However, it should be noted that, despite this, the various derivation methods perform similarly with regard to outcome prognostication (Liu et al., 2017a; Beqiri et al., 2023).

### Limits of reactivity

The upper limit of reactivity (ULR) and lower limit of reactivity (LLR) refer to the critical CPP thresholds beyond which CVR becomes compromised. These limits can be identified by plotting the U-shaped CPP vs. CVR relationship and identifying the CPP values at which the curve crosses a predefined CVR threshold, representing the transition point from intact to impaired reactivity. The CPP range between the LLR and ULR theoretically represents a range where CVR is intact. Up until this point, all studies on CPPopt considered only a single CPP target, ignoring the possibility that a broader CPP range could provide similar autoregulatory benefits.

In a 2017 study, Donnelly et al. investigated these limits of reactivity and their associations with long-term outcomes (Donnelly et al., 2017). The LLR and ULR were continuously calculated alongside CPPopt using a PRx threshold of +0.30. Time spent with CPP below the LLR was found to be strongly associated with both unfavorable outcome and mortality (p < 0.001). On the other hand, time spent with CPP greater than the ULR was only associated with unfavorable outcome (p < 0.02). Interestingly, while time spent with CPP more than 10 mmHg below CPPopt was strongly associated with increased mortality (p < 0.001), time spent with CPP more than 10 mmHg above CPPopt was not and instead exhibited an inverse relationship (p < 0.001). This suggests that maintaining CPP above CPPopt, provided it does not exceed the ULR, may not necessarily lead to worse outcomes. These findings support the use of a CPP range rather than a singular CPP target.

### Association with outcome

Overall, the relationship between CPPopt and long-term patient outcomes is well established. Studies have consistently demonstrated that larger deviations from CPPopt, as well as more time spent deviating from CPPopt, are strongly associated with poor long-term outcomes (Steiner et al., 2002; Aries et al., 2012a; Dias et al., 2015; Depreitere et al., 2014; Young et al., 2016; Liu et al., 2017a; Zeiler et al., 2019c; Lang et al., 2015; Riemann et al., 2020; Petkus et al., 2020). Additionally, CPPopt has been shown to display a more robust association with patient outcomes than current guideline-based CPP targets (Aries et al., 2012a). However, while there has been a generally unanimous agreement that spending time below CPPopt is associated with poor outcomes, there has been mixed evidence regarding the impact of spending time above CPPopt.

Some studies, including the original study by Steiner et al., found that both CPP above and below CPPopt are associated with mortality (Steiner et al., 2002; Depreitere et al., 2014), while others have suggested that hyperperfusion is only associated with severe disability and not mortality (Aries et al., 2012a; Liu et al., 2017a; Lang et al., 2015). Interestingly, there have been a handful of studies that have found that time with CPP above CPPopt is not associated with poorer outcomes (Young et al., 2016; Donnelly et al., 2017; Zeiler et al., 2019c; Stein et al., 2023; Svedung Wettervik et al., 2023a). A study by Stein et al. even found that % time spent with CPP above CPPopt was generally associated with improvement in long-term outcome (Stein et al., 2023). Similarly, a study by Petkus et al. found that, when CPPopt is within a range of 60–80 mmHg, maintaining CPP in a slightly hyperperfused state (up to 10 mmHg above CPPopt) was associated with better outcomes (Petkus et al., 2020).

The association between CPPopt and outcomes has also been demonstrated in pediatric populations. In 2018, Lo et al. found that there was significantly greater time spent with CPP near CPPopt among survivors and patients with favourable outcomes (p = 0.04 and p = 0.01, respectively) (Lo et al., 2018). Two later studies similarly found that time spent with CPP more than 10 mmHg below CPPopt was significantly associated with poorer outcomes (Svedung et al., 2024; Velle et al., 2023). Interestingly, a study by Lennell et al. found that, in contrast to younger patients, elderly patients do not demonstrate better outcomes when actual CPP is maintained near CPPopt (Lenell et al., 2024).

### Associations with physiology, demographics, and clinical factors

CPPopt has been shown to demonstrate associations with a wide variety of patient-specific factors. Older age has been shown to be associated with greater CPPopt values, suggesting that elderly patients may require greater cerebral perfusion in order to maintain intact CVR (Lenell et al., 2024; Young et al., 2021). Longer time since injury and presence of diffuse axonal injury have also been linked with higher CPPopt values (Young et al., 2021). Similarly, this may suggest that increased cerebral perfusion is needed to help maintain cerebral protective measures in patients in the chronic recovery phase or who have more global injury. CPPopt yield has also been shown to be affected by various clinical, physiologic, and demographic factors. The absence of slow ABP waves, greater PRx, lower dose of sedative/analgesic drugs, higher dose of vasopressor drugs, absence of neuromuscular blockers, and having had a decompressive craniectomy have all been identified as independent predictors for the absence of a distinct CPPopt curve (Weersink et al., 2015). It has also been suggested that short term moderate hypocapnia contributes to improved CPPopt identification (Haubrich et al., 2011). The following factors have been found to be not associated with CPPopt yield: age, admission Glasgow Coma Scale (GCS), gender, pupil response, prehospital hypoxia or hypotension, Marshall computed tomography (CT) score, decompressive craniectomy, injury severity score, or 24-h therapeutic intensity level (TIL) score (Liberti et al., 2021).

Having actual CPP below CPPopt has been shown to be associated with reduced CBF (Johnson et al., 2017). Similarly, blood pressure variability is strongly related to deviation from CPPopt (Svedung et al., 2020). Brain tissue oxygenation (PbtO_2_) levels have also been associated with deviation from CPPopt (Svedung Wettervik et al., 2023b; Megjhani et al., 2023). Interestingly, a nonlinear relationship between PbtO_2_ and ΔCPPopt (p < 0.001) has been observed, where PbtO_2_ decreases when ΔCPPopt was negative (CPP below CPPopt), but remained stable when ΔCPPopt was positive (CPP above CPPopt) (Megjhani et al., 2023). This suggests that hypoperfusion is more detrimental to oxygenation than mild hyperperfusion. An important question is if CPPopt targeted care is actually associated with more intact CVR. Beqiri et al. attempted to answer this question in a 2024 sub analysis of the COGiTATE study (Beqiri et al., 2024). The authors found that, in the intervention group, PRx was lower when CPP remained within ±5 mmHg of CPPopt (p < 0.001), but only when the PRx at the identified CPPopt was negative. This raises concerns about current CPPopt methodology, as it identifies a CPPopt as long as its associated PRx value is less than +0.60 (Beqiri et al., 2023).

The impact of CPPopt deviations on cerebral metabolism has been explored across different patient populations. In severe TBI patients, Wettervik et al. found that time spent within 10 mmHg of CPPopt was associated with lower cerebral lactate/pyruvate ratio (LPR) and lower glycerol levels, suggesting that maintaining CPP within an optimal range preserves metabolic homeostasis (Svedung et al., 2021). However, the metabolic effects of CPPopt deviations may vary by pathology, as Wettervik et al. also found that deviations from CPPopt (both above and below) were not significantly associated with impaired cerebral energy metabolism in an aneurysmal SAH cohort, as measured by microdialysis-derived glucose, LPR, and pyruvate levels (Svedung et al., 2022). In pediatric TBI, Velle et al. found that when ΔCPPopt exceeded +10 mmHg, lactate (p = 0.026) and LPR (p = 0.002) were significantly higher than when ΔCPPopt was below −10 mmHg (Velle et al., 2024).

### Use of alternative CVR indices for CPPopt derivation

Though much of the CPPopt literature has relied on PRx for derivation, other CVR indices can theoretically be used. Currently, no definite conclusions have been made on which index is best suited for deriving CPPopt; however, several studies have assessed the utility of various indices.

In 2011, Santos et al. investigated the use of low-frequency PRx (L-PRx) for deriving CPPopt (Santos et al., 2011). While PRx requires high-frequency waveform physiology, L-PRx is able to assess CVR using only minute resolution ICP and MAP data. The ability to calculate CPPopt using lower frequency data would make calculating this personalized metric more feasible for sites where high-frequency physiology monitoring is not available. Using a cohort of ICH patients, the authors found that CPPopt derived using L-PRx correlated very strongly with CPPopt derived using PRx (r = 0.980, p < 0.001), and even found that deviation yield was slightly higher for L-PRx-based CPPopt. However, in a TBI cohort, Lang et al. found that, while CPPopt values derived using PRx and L-PRx were quite similar, only PRx-based CPPopt demonstrated a statistically significant association with mortality and morbidity (Lang et al., 2015). A later study by Riemann et al. was able to demonstrate an association between L-PRx-based CPPopt and outcome, but found that it did not display the discriminative capacity of its high-resolution counterpart (Riemann et al., 2020).

In 2017, Liu et al. introduced a novel method for assessing CVR through the development of a wavelet transform-based PRx (wPRx) (Liu et al., 2017b). Unlike PRx, which uses a simple Pearson correlation, wPRx applies a wavelet transform phase shift between ABP and ICP, making it more resistant to signal noise and more temporally stable. This index has demonstrated a higher temporal stability and stronger association with patient outcomes than compared to PRx. It was found that CPPopt derived using wPRx and PRx correlated well (r = 0.81, p < 0.001); however, wPRx-based CPPopt displayed significantly greater yield (59.6% ± 27% vs. 53.2% ± 20%, p < 0.001) and stability (patient SD of 7.05 ± 3.78 vs. 8.45 ± 2.90, p < 0.001). wPRx and PRx-based ΔCPPopt displayed similar associations with patient outcomes.

Next, Zeiler et al. conducted a study in 2019 comparing the outcome associations of CPPopt derived using PRx, PAx, and RAC (Zeiler et al., 2019c). PRx- and RAC-based CPPopt were found to display similar associations with long-term patient outcomes, with RAC trending toward slightly higher AUC values. In contrast, PAx-based CPPopt failed to demonstrate any statistically significant associations with mortality or morbidity, raising questions about its utility in CPPopt derivation. In a later study, Lilja-Cyron et al. found somewhat differing findings (Lilja-Cyron et al., 2021). While time spent below CPPopt was associated with outcome upon univariate analysis for all three ICP-based indices, only dose of CPP below PRx-based CPPopt was associated with outcome. Furthermore, upon multivariable logistic regression analysis, only time/dose of CPP below PRx-based CPPopt was able to add any significant prediction capability to the baseline multivariable model.

Other CVR indices, not based on ICP, have also been investigated for their role in deriving CPPopt. For example, CPPopt derivation has been shown to be feasible with COx, the brain tissue oxygenation index (ORx), the cerebral blood flow index (CBFx), and TCD-based Mx and Sx (Dias et al., 2015; Zeiler et al., 2018c). However, only a moderate level of agreement has been demonstrated between PRx- and Mx-based CPPopt (Pochard et al., 2021), and no correlation was found between ORx- and PRx-based CPPopt (Radolovich et al., 2009). NIRS-based indices have also been explored. For example, one study derived CPPopt using the total hemoglobin reactivity index (THx), calculated as the correlation between MAP and NIRS-based total hemoglobin, and found significant correlation between it and PRx-based CPPopt (r = 0.74, p < 0.0001) (Zweifel et al., 2010).

### Current limitations and future directions

Despite the substantial amount of research that has been conducted on the concept of CPPopt, making it the most extensively studied personalized cerebral physiologic target to date, there remain several noteworthy limitations that need to be addressed. One major limitation in the current literature is the incomplete documentation of the algorithms used to derive CPPopt. While these algorithms are integrated into the ICM + software, making CPPopt derivation widely accessible, their detailed methodology remains proprietary and generally unavailable to the public. This lack of transparency limits opportunities for further refinement and validation by other lab groups. Although recent work by Van Twist et al. recently introduced an open-source CPPopt algorithm (van Twist et al., 2024), additional efforts are needed in order to allow for wider accessibility in enhancing algorithmic accuracy and yields.

Another issue that has been raised is the potential bias in CPPopt estimation due to the nature of cross-correlated signals. In 2018, Kelly et al. demonstrated that a U-shaped distribution between CPP and PRx can appear even when ICP and MAP signals are randomly generated (Kelly et al., 2018). This suggests that even if the underlying data does not represent actual CVR, a U-shaped relationship may still be observed due to statistical bias. This notion raises concerns that CPPopt values may not always be physiologically meaningful without proper statistical correction. While techniques such as Fisher transformation have been shown to help address this bias (Kelly et al., 2018), most CPPopt studies have failed to apply such corrective techniques. To improve the reliability of CPPopt as a personalized physiologic metric, future studies should incorporate correction techniques to ensure that any observed patterns truly reflect CVR rather than random statistical biases.

Next, there remains no definite conclusions on which CVR index is most ideal for deriving CPPopt. Although there have been a handful of studies that have compared some CVR indices to a limited capacity (Santos et al., 2011; Dias et al., 2015; Zeiler et al., 2019c; Lang et al., 2015; Riemann et al., 2020; Liu et al., 2017b; Lilja-Cyron et al., 2021), a comprehensive study comparing a wide range of indices for deriving CPPopt is needed. Various factors should be considered in comparing indices, such as derivation yield, CPPopt stability, association with outcome, and association with secondary brain injury metrics.

While observational studies have consistently shown that deviations from CPPopt are associated with worse outcomes, causality remains unproven. Before CPPopt-targeted management can be widely adopted, a randomized control trial is needed to demonstrate its efficacy. This has prompted the creation of the CPPopt Guided Therapy: Assessment of Target Effectiveness (COGiTATE) study, a phase II feasibility trial (Beqiri et al., 2019). This trial recently concluded, demonstrating that CPP-targeted management is both feasible and safe in TBI patients requiring ICP management (Tas et al., 2021). However, this study also found that the proportion of time spent with CPP within the CPPopt range was similar irrespective of whether it was targeted or not, thus raising questions about the necessity of precisely targeting CPPopt. This study has set the stage for a phase III CPPopt efficacy trial to formally determine whether CPPopt-targeted management results in improved long-term functional outcomes compared to current guideline-guided management.

Another natural question that remains is how aggressively CPP should be maintained near CPPopt. Whether maintaining CPP within 5 mmHg of CPPopt is necessary, or if keeping it within 10 mmHg is sufficient, is still unknown. Additionally, one wonders of the relative benefit of targeting a CPPopt target versus using the LLR as a CPP threshold. The current uncertainty around the importance of avoiding CPP hyperperfusion questions how maintaining CPP close to CPPopt compares to just maintaining CPP above the LLR. Future work is needed comparing CPPopt- and LLR-guided management of CPP.

## Mean arterial pressure optimum (MAPopt)

In 2001, Czosnyka et al. explored the relationship between CVR and cerebral physiology and found that a U-shaped relationship between MAP and Mx existed, indicating that both excessively low and high MAP were associated with impaired CVR (Czosnyka et al., 2001). This finding, along with evidence of significant inter-individual variability in the LLR (Brady et al., 2010b; Brady K. et al., 2010), suggested that fixed MAP thresholds may not be ideal. Instead, patient-specific MAP targets, that optimize CVR, could be more beneficial in preventing cerebral hypoperfusion and hyperperfusion.

**FIGURE 5:**
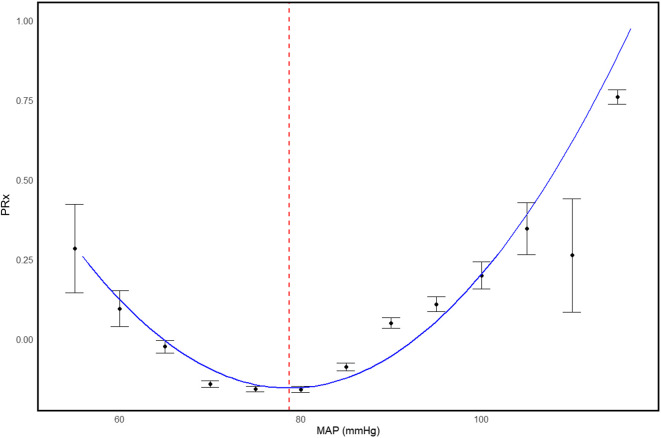
Patient example of MAPopt identification using an error bar plot and 5 mmHg MAP bins. Error bars represent 95% confidence intervals. The blue curve represents the fit parabolic curve. The vertical red dashed line represents the identified MAPopt value. PRx, pressure reactivity index; MAP, mean arterial pressure; MAPopt, mean arterial pressure optimum; mmHg, millimeters of mercury. University of Manitoba Health Research Ethics Board approval for generation of this image - H2024:266.

In 2010, Zweifel et al. highlighted that while PRx is valuable for deriving CPPopt, its reliance on invasive ICP monitoring limits its utilization for determining a personalized MAP target (Zweifel et al., 2010). The authors, therefore, investigated whether NIRS-based THx could be used to derive an optimal MAP target. Using the same fundamental principles underlying CPPopt identification, MAP was divided into 5 mmHg bins and the average THx, or PRx, was calculated for each bin. If a U-shaped relationship was observed, the MAP value at the nadir (lowest point) was identified as the MAPopt. A patient example of how MAPopt is identified can be found in Figure 5. It should be noted that in this work, and many other early works, the term “arterial blood pressure optimum” (ABPopt) was used; however, for the purposes of simplicity, we will use the term “MAPopt” throughout this review.

In a cohort of 40 head-injured patients, the authors identified MAPopt in 63.3% of patients when using PRx and 50% when using THx. A signed-rank test showed no statistical difference between MAPopt values derived from PRx and THx (p = 1.0), with a strong correlation observed between the two methods (r = 0.82, p < 0.0001). This study demonstrated the feasibility of deriving a patient-specific optimal MAP target using the relationship between MAP and CVR, and the possibility of doing so using a non-invasive CVR index.

Subsequent studies explored the clinical implications of this patient-specific MAP target. In 2013, Howlett et al. used the hemoglobin volume index (HVx) to derive MAPopt in neonates with hypoxic-ischemic encephalopathy (HIE) undergoing therapeutic hypothermia (Howlett et al., 2013). They found that maintaining MAP near or above MAPopt was associated with reduced neurologic injury, particularly in the paracentral gyri, white matter, basal ganglia, and thalamus. Additionally, those with minimal shifts in MAPopt between hypothermia and rewarming displayed better outcomes, suggesting that MAPopt stability may be predictive of lower injury severity. Two years later, Burton et al. supported these findings when they observed that neonates who suffered neurodevelopmental impairments at 2 years had higher MAPopt values and spent more time with MAP below MAPopt during rewarming (Burton et al., 2015). Interestingly, greater deviations above MAPopt were found to be associated with better cognitive outcomes.

In 2017, Lee et al. found that maintaining MAP near MAPopt during medically induced hypothermia was associated with lower creatinine levels in female neonates, suggesting a potential renal protective effect. Conversely, time spent with MAP below MAPopt during normothermia was correlated with elevated liver enzymes, indicating hepatic stress (Lee et al., 2017a). The authors also found that greater time spent below MAPopt was associated with brain injury and worse neurodevelopmental outcomes (Lee et al., 2017b). This suggests that targeting MAPopt could serve as an adjunct therapy alongside therapeutic hypothermia for neonatal HIE management as it may potentially provide organ protective effects.

MAPopt has been explored in post-cardiac arrest patients as well. In 2019, Sekhon et al. investigated the association between MAPopt and PbtO_2_ in hypoxic-ischemic brain injury following cardiac arrest (Sekhon et al., 2019). The authors used the Optimal Flex function found in ICM+, originally designed for CPPopt calculation, to derive MAPopt on a minute-by-minute basis. It was found that patients had better brain oxygenation when their MAP was within 5 mmHg of their MAPopt. When MAP was significantly below MAPopt, PbtO_2_ decreased; however, when MAP was above MAPopt, no further improvements in PbtO_2_ were observed.

Later that same year, Silverman et al. explored MAPopt in a cohort of aneurysmal SAH patients (Silverman et al., 2019). Using both PRx and the NIRS-based tissue oxygenation index (TOx), MAPopt was calculated on a minute-by-minute basis, along with the LLR and ULR, using a fixed 4-h sliding window. MAPopt could be identified in approximately 89.53% (±6.69%) of the total NIRS recording period, with a strong correlation between ICP- and NIRS-derived MAPopt observed. It was found time spent outside of the autoregulatory range (between the LLR and ULR) was significantly associated with worse outcomes at discharge and 90 days post-injury (p < 0.01). Each 10% increase in time spent outside this range was associated with a 2.8-fold increased risk of worse functional outcome. Additionally, patients with worse functional outcomes were interestingly found to have narrower autoregulatory ranges.

In 2020, Hoiland et al. conducted a study comparing PRx- and NIRS-based COx for MAPopt determination in hypoxic ischemic brain injury patients (Hoiland et al., 2020). The study found limited agreement between COx- and PRx-based MAPopt (mean bias: 1.4 mmHg; upper limit of agreement: 25.9 mmHg; lower limit of agreement: −23.0 mmHg), raising concerns about the reliability of NIRS-based indices for deriving MAPopt. However, when Oshorov et al. conducted a similar study in a cohort of severe TBI patients, good agreement between COx- and PRx-based MAPopt (bias = 0.39 ± 7.89 mmHg) was observed, with moderate correlation between the two (r = 0.49, p < 0.038) (Oshorov et al., 2022).

In 2021, Liu et al. further refined our understanding of MAPopt’s role in neonates with HIE (Liu et al., 2021). Using a novel wavelet hemoglobin volume index (wHVx), the study aimed to determine whether MAP near MAPopt was associated with less brain injury on MRI. Their results suggested that MAP exceeding MAPopt was correlated with reduced injury in critical brain regions such as the paracentral gyri, basal ganglia, thalamus, and brainstem. The study concluded that wavelet-based CVR monitoring may improve the identification of MAPopt in neonates and aid in developing individualized hemodynamic targets for improved neurological outcomes.

Most recently, Hazenberg et al. aimed to assess whether NIRS-based MAPopt measurements differ between left and right-sided recordings in comatose patients with hypoxic-ischemic brain injury after cardiac arrest (Hazenberg et al., 2023). Using COx_a from bifrontal rSO_2_ monitoring, MAPopt was calculated by leveraging a multi-window weighted approach. Results showed no significant differences in MAPopt values between left (80 mmHg, 95% CI: 76–84) and right (82 mmHg, 95% CI: 75–84) recordings (p = 1.0), with high correlation (0.95, p < 0.001). These findings suggest that unilateral NIRS recordings may be sufficient NIRS-based MAPopt determination in hypoxic-ischemic brain injury patients.

### Current limitations and future directions

Despite significant advancements in MAPopt research and its potential for individualized hemodynamic management in neurocritical care, several limitations remain. Firstly, there has yet to be substantial work on the development of an algorithm specifically tailored for MAPopt derivation. Currently, most studies have applied algorithms originally designed for CPPopt calculation. Although the same general principles underly both MAPopt and CPPopt identification, it is likely that they require different considerations for ideal derivation. Therefore, a dedicated MAPopt algorithm could enhance derivation accuracy and yield.

Next, further research is needed to explore factors influencing MAPopt derivation yield and stability. Sub-group analyses are needed in order to identify patient-specific factors that contribute to yield variability. Such work can help researchers further improve algorithmic derivation of MAPopt. MAPopt stability is also incredibly important. Currently, there is little to no evidence describing the stability (variability) in MAPopt outputs over time. Work that extensively evaluates and compares this parameter between various derivation methods is needed. This would allow for improvements in algorithmic stability.

The current literature on MAPopt application in the neurocritical care setting remains limited. Additionally, most of the studies performed on neurocritical patients have focused on the pediatric HIE population. Expanding the research in other neurocritical populations, particularly TBI, and other age cohorts is essential. These studies will need to establish associations between MAPopt, clinical outcomes, and cerebral physiology in these populations to demonstrate the potential clinical utility of MAPopt. Ultimately, randomized controlled trials will be required to establish the efficacy of MAPopt-guided therapy and its integration into standard neurocritical care protocols.

## Individualized intracranial pressure (iICP) thresholds

**FIGURE 6:**
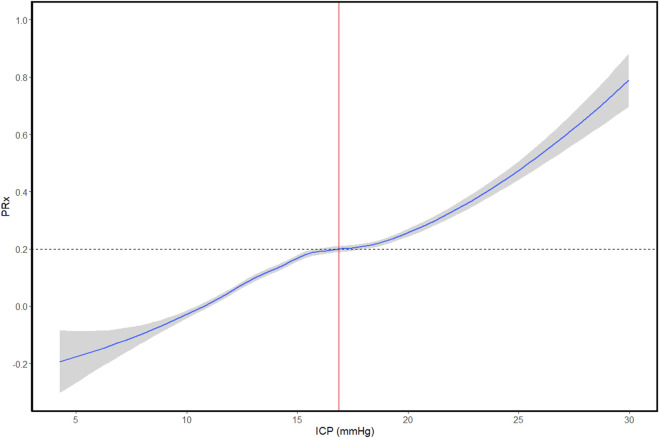
Patient example of iICP identification using a LOESS function. The blue line represents the LOESS curve. The grey region around the LOESS curve represents the 95% confidence interval. The vertical red dashed line represents the identified iICP value. The horizontal dotted black line represents the PRx threshold (PRx > 0.20) used to mark the transition between intact (below) and deranged (above) cerebrovascular reactivity. iICP, individualized intracranial pressure threshold; ICP, intracranial pressure; LOESS, locally weighted scatterplot smoothing; mmHg, millimeters of mercury; PRx, pressure reactivity index. University of Manitoba Health Research Ethics Board approval for generation of this image - H2024:266.

Unlike the U-shaped relationship seen with both CPP and MAP, ICP demonstrates a linear-like relationship with CVR, with increased ICP being associated with more impaired CVR (Stein et al., 2024). This is likely due to its relationship with CPP, where increasing ICP progressively reduces CPP below the lower limit of autoregulation, resulting in a pressure-passive state and a corresponding linear deterioration in CVR. Drawing from the same fundamental principles underlying CPPopt and MAPopt, a patient-specific ICP threshold, past which CVR becomes persistently deranged, can be derived using the function intersectionality between ICP and CVR. A patient example of iICP derivation using a LOESS curve can be found in Figure 6.

The concept of iICP thresholds was first introduced by Lazaridis et al., in 2014 (Lazaridis et al., 2014). Using the entire recording periods of 322 moderate-to-severe TBI patients, the authors plotted ICP against PRx using error-box plots, binning data in 4 mmHg of ICP increments. Then, through manual inspection, the ICP value at which PRx surpassed a threshold of +0.20 was identified. This threshold was chosen based on previous work from their group that demonstrated an association with impaired CVR and increased mortality (Czosnyka et al., 1997; Steiner et al., 2002). An iICP was only identified if a distinct transition from “intact” to “impaired” CVR, from PRx < +0.20 to persistently > +0.20, was observed. An iICP was identified in approximately 68% of patients, with a mean value of 25 ± 10 mmHg. Then, for each patient, the authors calculated the ICP “dose” (magnitude × time) spent above their determined iICP threshold, whenever possible, and guideline-based ICP thresholds to quantify insult burden. This metric takes both the magnitude and duration of all intracranial hypertensive episodes into account and was calculated as the cumulative area under the curve above threshold on the ICP time trend. Upon receiver operator curve (ROC) analysis, the authors found that ICP dose above iICP was a stronger predictor of outcome than dose above either guideline-based ICP threshold. Area under the receiver operator curves (AUC) and 95% CIs of 0.81 (0.74–0.87), 0.75 (0.68–0.81), and 0.77 (0.70–0.83) were calculated for iICP, ICP > 20 mmHg, and ICP > 25 mmHg, respectively.

The only other published work on ICP thresholds was a 2021 multi-center cohort validation study by Zeiler et al. (2021c). In this study, the authors were able to develop a semi-autonomous algorithm based on the methodology laid out in the previous study. For each patient, the algorithm created an error-bar plot, using 2.5 mmHg bins of ICP, and then applied a locally weighted scatterplot smoothing (LOESS) function to smooth the data. The intersection between the LOESS curve and a PRx threshold of > +0.20 was then identified as the iICP. The accuracy of the algorithm was then assessed through manual inspection of each patient’s constructed plots, with any inaccuracies corrected by hand.

When used on a cohort of 196 patients form the CENTER-TBI database, the algorithm was able to identify an iICP in 65.3% of patients (mean = 23.0 ± 11.8 mmHg), with an accuracy of 83.2%. Upon Mann-Whitney U testing, comparing patients who had an identifiable iICP with those who did not, it was found that those without an identifiable iICP had a higher mean ICP (p = 0.041) and PRx (p < 0.0001). When dichotomizing patients based on 6-to-12-month outcome into alive vs. dead and favorable vs. unfavorable outcome, mean hourly dose above iICP was found to be significantly different between the groups (p = 0.010 for alive vs. dead, p = 0.020 for favorable vs. unfavorable), with greater dose associated with the poorer outcome groups. Mean hourly dose above 20 mmHg or 22 mmHg failed to present a statistically significant difference between outcome groups. Upon univariate logistic regression analysis, dose of ICP above iICP was more strongly associated with 6-to-12-month outcome (AUC = 0.678, p = 0.029 for alive/dead, and AUC = 0.610, p = 0.060 for favorable/unfavorable) than either dose above 20 mmHg (AUC = 0.509, p = 0.034 for alive/dead, and AUC = 0.463, p = 0.236 for favorable/unfavorable) or 22 mmHg (AUC = 0.492, p = 0.035 for alive/dead, and AUC = 0.463, p = 0.263 for favorable/unfavorable).

### Current limitations and future directions

Though the findings from these preliminary studies have been promising, the current state of the iICP concept suffers from several limitations that prevent clinical applicability. First, the original methodology for deriving iICP relied on manual inspection of the iICP versus PRx plot (Lazaridis et al., 2014). This can be quite labor intensive, requiring trained personnel and introducing a significant amount of interpersonal variability. Though Zeiler et al. were able to develop a semi-automated approach, manual inspection is still required for validation (Zeiler et al., 2021c). A fully automated algorithm, which can derive iICP with high accuracy, is needed to address this limitation. Furthermore, both studies derived iICP using patients’ entire recording periods. This means that, in its current form, iICP can only be derived retrospectively after data collection is completed. This prevents its use in guiding therapeutic decisions in real-time during the early, critical stages of a patient’s ICU stay. A continuous derivation method is required to make clinical application feasible.

Next, the current derivation methods do not provide assessments of the quality of the output values, as not all iICP calculations are equal. The quality of the ICP versus CVR curve can vary drastically between data sets in multiple regards, such as curve shape, fit, CI widths, and data range. This creates a degree of uncertainty for the potential clinical end-user when presented with an iICP output. To address this, future renditions of iICP should include a robust curve grading system, enabling clinical end-users to gauge the quality of the iICP output value. Additionally, curve grading should be used to increase the quality of output values through use of a multi-window weighted technique, such as seen in the current CPPopt method (Beqiri et al., 2021).

Another limitation of the iICP concept is that both studies have relied exclusively on PRx to derive iICP. This raises the question of how other CVR indices compare in deriving iICP, especially considering that recent literature suggests that AMP-based indices, such as PAx and RAC, may be more strongly associated with long term outcomes compared to PRx in certain sub-group populations (Zeiler et al., 2018a; Zeiler et al., 2019b; Aries et al., 2012b). Additionally, both studies used an arbitrary threshold of +0.20. Using other thresholds would likely influence iICP derivation yield and its ability to predict outcome. Therefore, a systematic analysis comparing various CVR indices and thresholds is necessary to identify the combinations that produce the best performing iICP thresholds.

Though preliminary outcome associations have been assessed, further work will be needed to evaluate association between continuously derived iICP and long-term outcomes. Furthermore, the relationship between iICP and multi-modal cerebral physiology has yet to be evaluated. This is needed to better understand the utility of iICP in reducing secondary brain insult. Finally, the two existing studies were only able to identify an iICP in approximately 67% of patients (Lazaridis et al., 2014; Zeiler et al., 2021c). Various factors likely influence this yield, such as the CVR index and threshold used for derivation. Future work exploring the relationship between derivation yields and patient-specific factors, such as demographics, injury characteristics, and treatment modalities, is needed to better understand the determinants of derivation yields.

## Bispectral index optimal (BISopt)

Sedatives are routinely used in the neurocritical care setting to help prevent agitation, which can cause elevations in ICP, and reduce metabolic activity, which is thought to help preserve neural tissues in the acute phase of injury (Carney et al., 2017; Rangel-Castilla et al., 2008a). However, excessive sedation may suppress neurological responsiveness, potentially masking important clinical signs and delaying identification of evolving secondary brain injury mechanisms. Moreover, excessive sedation has been shown to be associated with poor long-term cognitive outcomes in critically ill patients (Girard, 2018; Porhomayon et al., 2016; Stephens et al., 2018). Therefore, monitoring a patient’s depth of sedation is vital for properly titrating sedatives to balance the benefits and risks associated with their administration. In the clinical setting, depth of sedation is generally measured using the Richmond Agitation Sedation Scale (RASS) (Ely et al., 2003; Sessler et al., 2002). This clinical grading system is performed by assessing a patient’s response to verbal or physical stimuli and assigning a score ranging from −5 (unarousable) to +4 (combative), with a score of 0 indicating a calm, alert patient. RASS is quick, inexpensive, and relatively easy to use; however, despite its widespread use, RASS suffers from several significant limitations.

Firstly, it is highly subjective, resulting in a high degree of interobserver variability. Second, it is not suitable for use on patients with impaired vision, hearing, somatosensation, or motor function since one cannot accurately assess such a patient’s response to verbal and physical stimuli. RASS is also limited by the fact that it only assesses observable responses to stimuli and, therefore, does not provide insight into the neurological activity or hemodynamic state of the brain. A recent study by Park et al. demonstrated that RASS is not statistically associated with measures of cerebral physiologic insult burden and that significant variability of these measures exists within each RASS category (Park et al., 2024). Finally, RASS only allows for single time-point measurements and thus cannot be used to continuously measure a patient’s depth of sedation.

The Bispectral Index (BIS), derived using electroencephalography (EEG), is a widely recognized method of quantifying a patient’s level of consciousness (Gan et al., 1997; Mathur et al., 2024). It provides a dimensionless numerical score ranging from 0 (complete suppression of brain activity) to 100 (fully awake), with values between 0 and 20 indicating EEG burst suppression (Mathur et al., 2024; Mitchell-Hines et al., 2016). This monitoring modality enables objective evaluation of one’s depth of sedation and its dose-response relationship with sedative/anesthetic administration. This parameter has been shown to correlate well with RASS and other clinical sedation scores (Deogaonkar et al., 2004); however, unlike these scores, BIS offers a more precise, quantifiable, objective measure of depth of sedation that can be measured continuously.

**FIGURE 7:**
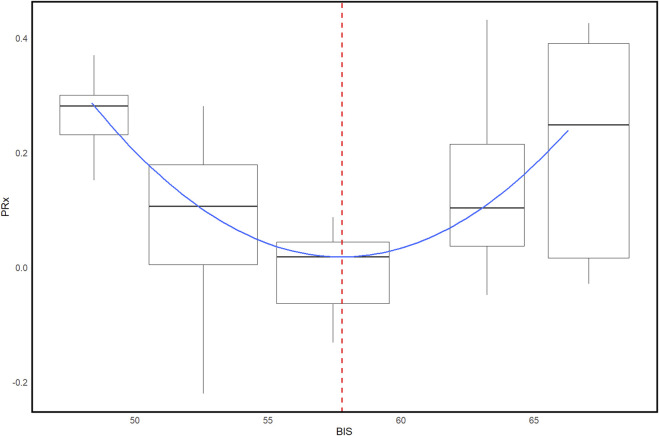
Patient example of BISopt identification using a box plot and 5 mmHg BIS bins. Box plots illustrate the median, IRQ, and minimum/maximum values of the bin. The blue curve represents the fit parabolic curve. The vertical red dashed line represents the identified BISopt value. BIS, bispectral index; BISopt, bispectral index optimum; mmHg, millimeters of mercury; PRx, pressure reactivity index. University of Manitoba Health Research Ethics Board approval for generation of this image - H2024:266.

It has been shown that a great deal of heterogeneity in the dose-response to sedative agents exists between patients (Froese et al., 2020b; Froese et al., 2020c; Zeiler et al., 2016). This limits the value of dosing information provided with sedative agents and raises questions on the optimal amount of sedative to administer to a patient. In a 2021 case series, which included five adult moderate-to-severe TBI patients, Froese et al. investigated the high-frequency relationship between BIS and CVR (Froese et al., 2021b). When plotting PRx versus BIS using error-bars, the authors observed a parabolic relationship between the two variables, similar to that seen between PRx and CPP. This suggests that both under- and over-sedation can expose moderate-to-severe TBI patients to derangements in CVR. Based on this observation, the authors speculated that an optimal depth of sedation for preserving CVR may be possible to calculate by identifying the BIS value at which PRx is minimized (most intact). An example of BISopt derivation can be seen in Figure 7. Additionally, using data from two patients with more than 12 h of uninterrupted BIS and PRx data, the authors demonstrated the feasibility of deriving such an optimal depth of sedation continuously by calculating values for consecutive 4-h, non-overlapping windows of data.

One year later, the same group further investigated the concept of a BIS-based optimal depth of sedation, which they termed BISopt (Froese et al., 2022a). Using the entire recording periods of 32 patients, Froese et al. were able to identify, through manual inspection, BISopt in 84.4% of cases for left-sided BIS and 71.9% for right-sided BIS. The authors then created a BISopt algorithm by leveraging an automated quadrative curve fitting method and minute-by-minute BIS/PRx data. In short, this algorithm divides the data into 3 a.u. BIS bins, creates a boxplot, and fits a second-order polynomial function to the plot. If the fitted curve fulfills a list of criteria, the minimum point of the curve is identified as the BISopt value. In some cases, only a descending or ascending portion of a curve is observed. In these cases, if a positive convex shape is maintained, BISopt is still identified but likely over or under-estimates the true value, respectively.

When applied to the entire recording periods of the 32 patient datasets, BISopt derivation yields were found to be 52.1% and 54.1%, for left- and right-sided BIS, respectively. The authors then compared their custom algorithm to the multi-window weighted Optimal Flex method, which was originally designed for calculating CPPopt and is included in the ICM + software (Depreitere et al., 2014). The Optimal Flex method displayed poorer yields, at 31.2% and 33.5%, for left- and right-sided BIS, respectively, and produced more inaccuracies (Froese et al., 2022a). This suggests that, despite CPP and BIS demonstrating similar parabolic relationships with CVR, CPPopt algorithms are not necessarily applicable to deriving BISopt.

The authors also derived continuous time trends of BISopt using their custom algorithm, by employing a sliding 4-h window that updates every minute. It was found that the optimal depth of sedation changes over the course of a patient’s ICU stay. Percent yields varied greatly between patients, and the authors found that the sedative and vasopressor, or combination, used has little effect on % yield. However, it was found that high levels of sedation were associated with a small increase in BISopt derivation yield. The association between BISopt and cerebral physiologic measures was also conducted. It was found that continuous BISopt demonstrated no significant associations with minute-resolution ICP, MAP, or CPPopt. This demonstrates that BISopt is likely a distinct physiologic metric that may allow for modulation of cerebral physiologic injury, independent from CPPopt.

In addition to the moderate-to-severe TBI population, BISopt may have a potential role in the management of general ICU patients, as multiple studies have demonstrated an association between sedation levels and outcomes in this population, as well as the presence of impaired CVR during the acute phase (Girard, 2018; Porhomayon et al., 2016; Stephens et al., 2018). However, the use of PRx, which requires invasive ICP monitoring for its derivation, limits the applicability of this personalized physiologic target to the general ICU population. To address this limitation, Froese et al. conducted another study in 2022 where they evaluated the feasibility of deriving BISopt using non-invasive COx_a instead of PRx (Froese et al., 2022b).

Using the same methodology as in their previous BISopt paper (Froese et al., 2022a), the authors derived BISopt using entire recording periods as well as continuously, but with COx_a rather than PRx (Froese et al., 2022b). When BISopt calculations were conducted on a cohort of 42 patients, Froese et al. observed that BIS demonstrates a similar parabolic relationship with COx_a as it does with PRx. When BISopt derived using COx_a was compared to BISopt derived using PRx, it was found that no statistically significant difference existed between the two with regard to median values (48 [IQR: 40–56] vs. 45 [IQR: 40–56], p = 0.31 upon Wilcox signed-ranked test). Additionally, yields were found to be somewhat similar to those seen with PRx-derived BISopt. Further, it was found that yields were generally independent of sedative or vasopressor used, as was seen with PRx-derived BISopt.

Until recently, BISopt had only been demonstrated in the neural injury population, and it was unknown whether this physiological target exists in healthy individuals. However, a recent study by Froese et al. aimed to validate the presence of BISopt in a non-neural injury population undergoing general anesthesia and confirm its absence in healthy, awake volunteers (Froese et al., 2024). The study found that BISopt was present in 96% of elective surgery patients under general anesthesia, suggesting that BISopt may also have potential a role in non-neural injury populations. Conversely, BISopt was found to be absent in fully awake volunteers, likely due to limited BIS variability and relatively stable CVR. This confirms that BISopt represents a physiologically meaningful target rather than merely statistical anomaly.

### Current limitations and future directions

Despite the promising work and potential applications of BISopt in managing sedation in the neurocritical care setting, the concept remains in its early stages and is subject to several limitations that hinder its clinical implementation. Firstly, it remains unknown whether this personalized depth of sedation measure is associated with patient outcomes. Therefore, a comprehensive outcome association analysis that evaluates whether maintaining BIS near BISopt (i.e., within ±5 a.u.) is associated with improved long-term outcomes is needed. Failure to demonstrate such associations would strongly question the clinical utility of this measure.

In addition to outcome associations, the relationship between BISopt and multi-modal cerebral physiology requires further exploration. Though Froese et al. demonstrated that continuous BISopt is not significantly associated with ICP, MAP, or CPPopt (Froese et al., 2022a), it remains poorly understood how BISopt influences the cerebral environment. This can be addressed thorough a comprehensive analysis exploring the associations between time spent with BIS near BISopt and various measures of cerebral physiology, such as cerebral compliance, CVR, and brain oxygenation. Such work could yield valuable insights into the potential role of BISopt in minimizing secondary brain injury.

Next, while Froese et al. were able to create a semi-automated algorithm for the derivation of BISopt, there remains a lot to be desired. Various methodologies, such as different windowing techniques and CVR indices, should be explored and compared to improve derivation yields and output accuracy. For example, the current algorithm leverages a fixed 4-h sliding window approach to derive BISopt continuously; however, the use of more sophisticated windowing techniques, such as the multi-window weighted methodology seen with recent iterations of CPPopt (Depreitere et al., 2014), may improve derivation yields.

Additionally, as it stands, the BISopt algorithm does not provide any quality assessments for its output values. This represents a critical gap, as the quality of the generated curve between BIS and CVR can vary significantly in terms of curve shape, curve fit, and data quality. For example, often only part of a curve is observed (either ascending or descending) and a BISopt value is estimated rather than directly identified. The inclusion of a measure of curve quality would allow clinical end-users to better evaluate the reliability of a BISopt value and help make more informed decisions when managing the sedation of critically ill patients.

Next, all four existing works on the topic of a personalized depth of sedation target have come from a single research group. Work from other groups will be needed to diversify and improve the reliability of the literature on the topic. Lastly, though Froese et al. disclosed the general principles that they used to derive BISopt, no openly accessible BISopt algorithm has been published. The absence of a publicly available algorithm limits the transparency of the current literature and acts as an obstacle for future work in this field by other lab groups. An open-access BISopt algorithm would not only promote transparency but also foster and accelerate further work on this promising personalized depth of sedation target.

## Conclusion

The exploration of personalized cerebral physiologic targets has opened a promising frontier in neurocritical care. These personalized targets offer a potential nuanced approach to patient care, aiming to tailor clinical management to a patient’s specific individual cerebral physiologic needs, and are increasingly recognized for their potential to guide therapeutic decisions and improve clinical outcomes in patients with neurocritical conditions. However, it is important to acknowledge the significant limitations that currently exist with these targets and the future work that is needed.

Firstly, most of the evidence supporting the use of personalized cerebral physiologic targets is based on highly curated datasets which do not reflect what is currently available at the patient bedside, where only basic heuristic filters are typically possible. Furthermore, the derivation of personalized targets is generally not feasible using raw signal data. This necessitates the development of an automated artifact management tool, which can provide thoroughly artifact clear raw signal data in real-time using a variety of methods, such as thresholding, time-series, Fourier/wavelet, and machine learning techniques.

Second, despite promising findings suggesting potential clinical benefit from the use of personalized physiologic targets, none of the personalized targets discussed in this review have been validated through large-scale interventional trials. Therefore, caution is advised when interpreting the existing literature as it remains currently unknown whether any of these personalized targets offer any real clinical utility. Future randomized control trials will be needed to confirm whether or not any of the use of personalized targets actually results in improved patient outcomes. Another limitation of the personalized targets is that the key assumptions underlying CVR indices, such as the notion that changes in pulsatile cerebral blood volume (i.e., ICP) are primarily driven by changes in systemic pressure, are frequently violated due to clinical interventions, neurovascular coupling, and external factors. This raises questions on whether these targets actually optimize a patient’s cerebral autoregulatory status.

**FIGURE 8:**
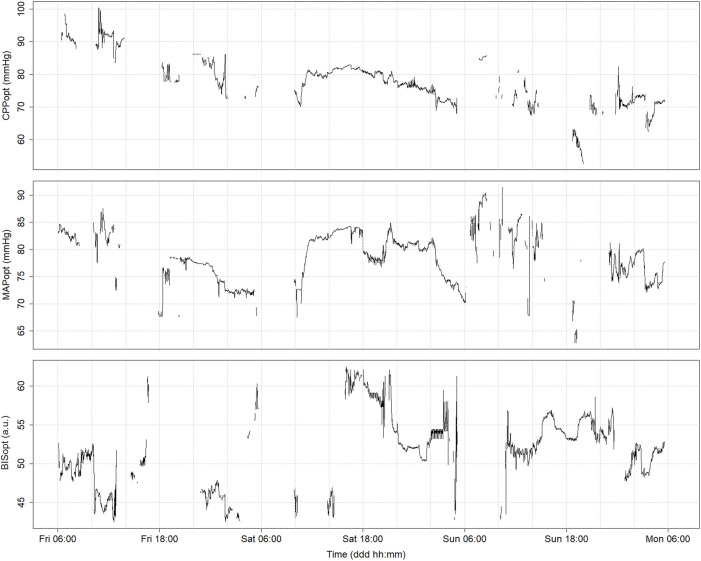
Time trends of continuously derived CPPopt, MAPopt and BISopt in tandem. Time trends display minute-by-minute data over a 72-h period. a.u., arbitrary units; BISopt, bispectral index optimum; CPPopt, cerebral perfusion pressure optimum; MAPopt, mean arterial pressure optimum; mmHg, millimeters of mercury. University of Manitoba Health Research Ethics Board approval for generation of this image - H2024:266.

Next, if personalized cerebral physiologic targets are to be deployed in neurocritical care setting, there must be efforts to integrate them into a multi-modal personalized medicine platform. An example of time trends for CPPopt, MAPopt, and BISopt in tandem can be found in Figure 8 (iICP is not presented here as no continuous iICP algorithm currently exists). A natural question will be how to manage conflicts between targets if they arise. It is currently not possible to provide an answer for this, as it remains unknown how these personalized targets compare in their influence on secondary brain injury reduction and how they influence each other. Therefore, future work will be needed to better understand the relationship between these physiologic targets, including their autocorrelative structures, and how to best tackle targeting the various targets in tandem.

Lastly, the derivation yields for the various personalized metrics remain unideal. This results in suboptimal continuity in signal, as seen in Figure 8. Things such as data gaps, caused by artifacts or recording errors, and limited physiologic variability can hinder identification of personalized targets. Future work will be needed to help tackle these shortcomings and improve derivation continuity. One potential avenue to address these issues is the use of machine learning. Leveraging advanced machine learning techniques may allow for data interpolation and identification of targets despite limited data variability. Deimantavicius et al. have already demonstrated the promise that such techniques offer. In a recent 2012 study, the authors were able to leverage a machine learning-based algorithm to improve the continuity and reliability of CPPopt estimates, and enable identification of valid CPPopt targets using shorter monitoring windows (Deimantavicius et al., 2022). Machine learning offers a promising way forward in the improvement of personalized cerebral physiologic targets.
